# Advancing Prenatal Diagnosis: From Conventional Karyotyping to Genome-Wide CNV Analysis

**DOI:** 10.3390/life16020309

**Published:** 2026-02-11

**Authors:** Elitsa Gyokova, Eleonora Hristova-Atanasova, Elizabeth Odumosu, Kamelia Dimitrova

**Affiliations:** 1 Department of Obstetrics and Gynecology, Faculty of Medicine, Medical University-Pleven, 5800 Pleven, Bulgaria; elitca.gaokova@mu-pleven.bg; 2 Clinic of Obstetrics and Gynecology, University Hospital “Saint Marina”, 5800 Pleven, Bulgaria; 3 Department of Social Medicine and Public Health, Faculty of Public Health, Medical University of Plovdiv, 4002 Plovdiv, Bulgaria; 4 Faculty of Medicine, Medical University-Pleven, 5800 Pleven, Bulgaria; elizabeth.odumosu@outlook.com (E.O.); k.kamenova.96@gmail.com (K.D.)

**Keywords:** prenatal diagnosis, chromosomal microarray analysis (CMA), copy number variations (CNV), whole-genome sequencing, invasive testing, fetal anomalies

## Abstract

Background: Advances in genome-wide DNA-based technologies have fundamentally transformed prenatal genetic diagnostics, enabling detection of clinically significant submicroscopic chromosomal abnormalities that are not identifiable by conventional cytogenetic methods. These developments have important implications for the diagnosis and management of pregnancies complicated by fetal structural abnormalities, as they enable more accurate etiological diagnosis, improved prognostic assessment, and more informed clinical decision-making and reproductive counselling. Methods: This narrative review synthesizes contemporary international evidence on prenatal genetic diagnostic approaches, including conventional karyotyping, chromosomal microarray analysis (CMA), and genome-wide sequencing technologies. The review focuses on diagnostic performance, clinical utility, ethical considerations, and implementation within diverse healthcare systems. Results: Accumulating evidence demonstrates that genome-wide approaches—particularly CMA and sequencing-based methods—provide a higher diagnostic yield in fetuses with structural anomalies, with an incremental yield of approximately 3–5% over conventional karyotyping. This is mainly due to their ability to detect pathogenic copy number variants below the cytogenetic resolution of karyotyping. These technologies improve etiological insight, enhance genotype–phenotype correlation, and support more precise prognostication and reproductive counselling, especially in pregnancies with fetal structural anomalies. Emerging sequencing platforms further expand the diagnostic spectrum by integrating copy number and sequence-level variant detection. Conclusions: Genome-wide Copy Number Variation (CNV) analysis represents a critical component of contemporary prenatal diagnostics and should be integrated into invasive prenatal testing pathways in accordance with international recommendations. Genome-wide approaches need robust counselling frameworks and equitable health policy implementation to spread. The expense, lack of required experience, and variation in healthcare infrastructure across locations make widespread deployment difficult.

## 1. Introduction

Prenatal genetic diagnosis is routinely used to support clinical decision-making during pregnancy, particularly when there is concern about fetal development or postnatal outcomes [1]. Despite substantial progress in prenatal screening programs and advances in fetal imaging, congenital anomalies remain a leading cause of perinatal morbidity and mortality. Data indicate that important genetic abnormalities continue to escape detection in a proportion of pregnancies [2].

Conventional cytogenetic testing, most commonly karyotyping, has long been the standard method for invasive prenatal diagnosis. Karyotyping allows for the assessment of chromosome number and the identification of large structural rearrangements and balanced abnormalities. It remains highly effective for detecting common aneuploidies; however, its limitations become apparent when fetal structural anomalies are present and cytogenetic results are normal. In such cases, a genetic cause is frequently still suspected. Evidence accumulated over the past two decades suggests that pathogenic copy number variants (CNVs), which are not detectable by routine karyotyping, contribute substantially to fetal malformations and adverse developmental outcomes [3,4,5,6].

To close this diagnostic gap, chromosomal microarray analysis (CMA) was introduced into clinical practice. Through the ability to detect submicroscopic CNVs across the entire genome, CMA offers information that traditional cytogenetic analysis cannot. According to several studies, CMA increases the overall diagnostic yield of invasive prenatal testing by producing clinically meaningful results in pregnancies with fetal structural anomalies and a normal karyotype [3,7].

The scope of prenatal genetic evaluation has been further expanded by more recent advancements in genome-wide DNA-based technologies. Without depending on predetermined targets or particular clinical suspicion, comprehensive genome-wide CNV analysis and sequencing-based methods enable a more unbiased evaluation of genomic variation [8]. Although their application varies greatly across healthcare systems, these techniques are increasingly integrated into diagnostic workflows.

The aim of this review was to critically evaluate the evidence on prenatal genetic diagnostic methods, with a particular focus on the clinical role of genome-wide CNV analysis across diverse healthcare settings, including Bulgaria.

## 2. Materials and Methods

This narrative review was developed using a structured narrative approach, informed by the framework described by Templier and Paré. The objective was to critically summarize and interpret available evidence on prenatal genetic diagnostic methods, with an emphasis on genome-wide CNV analysis and its clinical role across different healthcare settings, including Bulgaria. A narrative review design was chosen to facilitate an integrative synthesis of diverse evidence, encompassing clinical studies, professional guidelines, ethical considerations, and health system-level implementation challenges, which are inadequately addressed by systematic or scoping review frameworks. The review was performed using the SANRA standard for narrative reviews to improve transparency and methodological rigour.

A literature search was conducted in several electronic databases, including Web of Science, MEDLINE/PubMed, Google Scholar, and the Cochrane Library. Searches were performed using keywords related to prenatal diagnosis, CMA, CNVs, whole-genome sequencing (WGS), invasive prenatal testing, and fetal structural anomalies. The PubMed search strategy comprised the following query: (“prenatal diagnosis” [Title/Abstract] AND (“chromosomal microarray” OR “copy number variation” OR “CNV” OR “genome-wide sequencing”)) [Title/Abstract], limited to human studies published in English. Title and abstract screening preceded a comprehensive evaluation of full-text eligibility.

When necessary, MeSH terms were employed along with Boolean operators. The search strategy was designed to capture new clinical evidence, technological advancements, and updates to international guidelines by focusing on publications published between January 2010 and December 2025.

Peer-reviewed original research articles, systematic and narrative reviews, international clinical guidelines, and policy-related reports pertaining to prenatal genetic diagnostics in human populations were considered for inclusion. Animal studies, case reports, conference abstracts, and non-English publications were excluded. The decision to restrict the review to English-language literature was a practical choice, although it might have led to an under-representation of data from certain national or regional contexts.

Cohort studies, case–control studies, cross-sectional analyses, prospective observational studies, qualitative research, and systematic or scoping reviews were among the study designs considered. The methodological quality, applicability to clinical practice, and relevance to prenatal genetic diagnostics of the retrieved sources were assessed. The evidence was synthesized narratively and arranged thematically, with a focus on issues pertaining to healthcare system implementation, clinical utility, diagnostic performance, and ethical considerations. In the process of evidence synthesis, qualitative consideration was given to platform-specific analytical limitations, such as minimum CNV size thresholds, varying sensitivity for low-level mosaicism, and discrepancies in bioinformatic pipelines between array-based and sequencing-based technologies. These methodological considerations were considered while analysing stated diagnostic yields and clinical performance indicators.

When applicable, the results were interpreted within the framework of the Bulgarian healthcare system, accounting for financing arrangements, access to specialized genetic diagnostic services, and the national organization of prenatal care. It is important to recognise that CNVs may contribute to both monogenic or highly penetrant syndromes and complex situations where genetic susceptibility interacts with environmental and non-genetic variables, affecting interpretation in prenatal contexts.

## 3. Conventional Methods for Prenatal Cytogenetic Evaluation

Conventional cytogenetic methods have long represented the basis of prenatal genetic diagnosis. These techniques provide genome-wide information on chromosomal numbers and large-scale structural organisation and have shaped clinical practice for decades. The most popular methods among them remain karyotyping and fluorescence in situ hybridisation (FISH), which are still used as benchmarks to assess the performance of newer genome-wide technologies.

### 3.1. The Karyotype

Karyotyping is a traditional cytogenetic technique for assessing chromosome number, size, morphology, and general structural integrity. To detect numerical and structural chromosomal abnormalities, the technique entails culturing foetal cells obtained through invasive prenatal procedures, arresting dividing cells in metaphase, staining the chromosomes (usually with Giemsa), and microscopically examining the cells [9,10,11,12]. A typical human karyotype consists of 46 chromosomes arranged in 23 pairs.

Giemsa banding (G-banding), the most widely used staining method, creates a distinctive pattern of alternating light and dark bands along each chromosome. This reproducible banding pattern enables chromosome identification and facilitates the detection of numerical anomalies and significant structural rearrangements [11,12,13].

Because G-banding is performed on metaphase chromosomes, when chromatin is highly condensed and morphologically distinct, it allows for genome-wide analysis without prior assumptions regarding the location of a potential abnormality [12]. Professional organisations, including the American College of Medical Genetics and Genomics (ACMG), recognise G-band karyotyping as a standard cytogenetic method for detecting large-scale chromosomal abnormalities across a range of clinical settings [14].

The effective resolution of traditional G-banded karyotyping in routine prenatal practice is restricted to approximately 5–10 megabases (Mb). While larger deletions, duplications, and rearrangements can be reliably identified, chromosomal abnormalities smaller than this size are typically undetectable [14,15,16]. Although this figure varies based on chromosome morphology and banding quality, quantitative evaluations have revealed that the average resolution attained in standard prenatal karyotyping is nearly 9 Mb [16]. Prometaphase and prophase analysis are examples of high-resolution banding techniques that can increase resolution to about 3–5 Mb, but due to their limited availability and high technical requirements, these methods are not frequently used in clinical prenatal diagnostics [17].

Karyotyping is especially useful for identifying sex chromosome abnormalities and whole-chromosome aneuploidies, such as trisomy 21, trisomy 18, and trisomy 13. It also consistently detects significant structural rearrangements, including balanced and unbalanced translocations, inversions, substantial deletions and duplications, and marker chromosomes [17,18,19]. An important advantage of karyotyping is its unbiased, genome-wide nature, which allows the detection of unexpected abnormalities and balanced rearrangements that may be missed by targeted molecular techniques [4,17,19,20]. Evidence from large cohort studies and external quality assessment programmes continues to support its reliability for these categories of chromosomal abnormalities [17,20].

At the same time, the limitations of karyotyping are well known. The method cannot reliably detect submicroscopic CNVs below the 5–10 Mb resolution threshold, including pathogenic microdeletions and microduplications frequently associated with congenital anomalies, developmental delay, and neurodevelopmental disorders [4,21,22]. In addition, karyotyping requires viable, dividing cells and therefore depends on successful cell culture, which may take several days to two weeks for prenatal samples. This results in longer turnaround times compared with DNA-based techniques [23]. Professional guidelines emphasise that karyotyping alone is insufficient for the comprehensive detection of clinically relevant genomic imbalances [17]. Other practical limitations in the prenatal setting include dependence on culture quality, the risk of maternal cell contamination, and variable sensitivity for detecting tissue-limited mosaicism.

### 3.2. Fluorescence In Situ Hybridisation (FISH)

Fluorescence in situ hybridisation (FISH) is a targeted molecular cytogenetic technique that uses fluorescently labelled DNA probes to hybridise to complementary genomic sequences within interphase nuclei or metaphase chromosomes, enabling direct visualisation of specific chromosomal regions [24,25,26]. Unlike karyotyping, FISH allows for faster analysis because it does not require cell division and can be used on uncultured cells.

With locus-specific resolution that surpasses that of traditional karyotyping, FISH can identify a variety of chromosomal abnormalities, such as aneuploidies, deletions, duplications, amplifications, and translocations [24,27,28]. Specimen types including formalin-fixed paraffin-embedded tissue, fresh samples, and cultured cells can be used with this technique, which is popular in clinical genetics and oncology [25,27,28]. In prenatal diagnostics, FISH is most frequently used for rapid aneuploidy screening of chromosomes 13, 18, 21, X, and Y. Results are usually available within 24–48 h, which significantly shortens turnaround time compared to karyotyping [29,30,31].

Beyond rapid aneuploidy screening, FISH may also be used to detect submicroscopic deletions, duplications, and cryptic rearrangements at predefined loci. It is frequently applied to confirm suspected abnormalities or to clarify equivocal karyotype findings [24,32,33,34]. Multicolour and multiplex FISH techniques allow simultaneous interrogation of multiple loci in a single assay, improving diagnostic efficiency in selected clinical scenarios [27,33,34].

The principal limitation of FISH lies in its targeted, locus-specific design. Only genomic regions covered by the selected probes are analysed; abnormalities outside these regions remain undetected [24,35,36]. Consequently, FISH does not provide genome-wide information and cannot identify unexpected chromosomal imbalances. The ACMG therefore emphasises that FISH should be regarded as a complementary technique or a rapid screening tool rather than a substitute for comprehensive genome-wide analysis [36].

### 3.3. Summary of Analytical Capabilities and Limitations

The main analytical characteristics, clinical applications, and limitations of conventional cytogenetic methods used in prenatal diagnosis are summarised in Table 1.

### 3.4. Limitations of Conventional Cytogenetic Techniques

Although karyotyping and FISH remain important components of prenatal cytogenetic evaluation, their combined limitations restrict overall diagnostic yield. Karyotyping lacks the resolution required to detect submicroscopic CNVs, while FISH is confined to predefined genomic regions and does not allow for an unbiased assessment of the entire genome. As a result, a substantial proportion of pathogenic genomic imbalances associated with foetal structural anomalies and adverse developmental outcomes remain undetected when conventional cytogenetic methods are used in isolation [4,21,22]. These limitations have directly contributed to reduced diagnostic yield and have driven the progressive shift toward higher-resolution, genome-wide, DNA-based diagnostic approaches.

## 4. Advanced DNA-Based Technologies in Prenatal Genetics

Advanced DNA-based technologies have markedly expanded the scope of prenatal genetic diagnostics by enabling genome-wide analysis of chromosomal and genomic variation. In contrast to conventional cytogenetic methods, these approaches allow for the detection of submicroscopic CNVs and, when sequencing-based strategies are applied, single nucleotide variants (SNVs) and small insertions or deletions (indels). The analytical scope, strengths, and key limitations of these modern technologies are summarised in Table 2. The increased analytical resolution achieved with DNA-based methods has translated into a higher diagnostic yield, particularly in pregnancies complicated by foetal structural anomalies [37,38,39,40,41].

### 4.1. Chromosomal Microarray Analysis and Genome-Wide CNV Detection

CMA enables genome-wide detection of submicroscopic deletions and duplications, typically achieving an analytical resolution of approximately 50–100 kb. This represents a substantial improvement over routine karyotyping and explains the consistently higher diagnostic yield observed in prenatal cohorts. At the same time, CMA is not designed to detect most SNVs or small indels and therefore has limited utility for the diagnosis of monogenic disorders [39,40,41].

CMA is performed on extracted DNA obtained from chorionic villus sampling or amniotic fluid and does not depend on cell culture, which offers practical advantages. Nevertheless, important limitations remain. Balanced chromosomal rearrangements are not detected by CMA, and sensitivity for mosaicism and triploidy is platform dependent. For this reason, the complementary use of conventional cytogenetic methods is still maintained in selected clinical contexts [4,42,43,44].

In clinical practice, CMA is implemented using array comparative genomic hybridisation (aCGH), SNP-array technology (Single nucleotide polymorphism), or combined platforms. While all platforms reliably detect copy number gains and losses, SNP-based designs provide additional genotype-level information and allow for the identification of copy-neutral findings, such as regions of absence of heterozygosity (AOH) and patterns suggestive of uniparental disomy (UPD) or consanguinity. These findings are clinically relevant for the diagnosis of imprinting disorders and for the assessment of recessive disease risk. Platform-specific factors, including probe density, genomic coverage, and reporting thresholds, influence diagnostic performance and interpretation [43,44]. In prenatal diagnostics, increasing resolution improves CNV detection, but it also increases interpretative complexity, highlighting the importance of standardised reporting practices [45,46,47].

Sequencing-based approaches for genome-wide CNV detection, commonly referred to as low-pass genome sequencing (LP-GS), have emerged as alternatives to array-based platforms. These methods use read-depth analysis across the genome to detect aneuploidies and CNVs and have demonstrated diagnostic performance comparable to CMA. LP-GS may be particularly attractive in laboratories already structured around next-generation sequencing (NGS) workflows. Their clinical implementation requires validated bioinformatic pipelines and defined analytical thresholds [50,51,52,53,54].

### 4.2. Whole-Exome and Whole-Genome Sequencing in Prenatal Diagnosis

Whole-exome sequencing (WES) and whole-genome sequencing (WGS) further extend prenatal genetic diagnostics by enabling the detection of SNVs and small indels associated with monogenic disorders. In principle, WGS offers the most comprehensive single-assay genomic assessment, although its diagnostic performance depends on sequencing coverage and analytical pipelines.

Clinical studies suggest that WGS can identify CNVs detected by CMA while providing additional findings in foetuses with structural anomalies. Both WES and WGS, however, require careful implementation because of interpretative complexity and the need for robust counselling frameworks [55,56,57,58,59,60]. Professional position statements, including those from the International Society for Prenatal Diagnosis (ISPD), emphasise that genome-wide sequencing should be supported by experienced genetic counsellors [59].

### 4.3. Non-Invasive Prenatal Testing: Screening Performance and Diagnostic Boundaries

Cell-free DNA-based non-invasive prenatal testing (NIPT) is a screening approach for common aneuploidies. Despite its high performance, NIPT is not a diagnostic test. Its limitations are particularly relevant when foetal structural anomalies are identified, as clinically significant genetic abnormalities may still be present despite a negative screening result. Accordingly, in pregnancies with foetal anomalies, invasive diagnostic testing using genome-wide methods, such as CMA, remains the recommended pathway. Positive NIPT results also require confirmation through invasive diagnostic testing [61,62].

## 5. Clinical Indications for Genome-Wide CNV Testing

CNV testing has become an integral component of prenatal genetic diagnostics owing to its ability to detect pathogenic submicroscopic chromosomal imbalances that are not identifiable by conventional karyotyping. Evidence from large cohort studies, supported by international professional recommendations, consistently demonstrates that clinically significant CNVs contribute substantially to foetal structural anomalies and adverse pregnancy outcomes across a range of prenatal risk scenarios [5,6,63].

### 5.1. Indications Across Prenatal Risk Settings

Foetal structural abnormalities detected by prenatal ultrasound represent the most robust and consistently supported indication for genome-wide CNV testing. Cohort studies and systematic analyses have shown that pathogenic or likely pathogenic CNVs are frequently identified in this context, even when conventional karyotyping yields normal results [5,6]. Across studies, CMA detects pathogenic CNVs in approximately 3–13% of foetuses with structural anomalies, with most reported yields clustering between 4% and 10%. Diagnostic yield is highest in foetuses with multiple or complex anomalies and in selected organ systems, particularly cardiac and multisystem malformations [64,65].

The diagnostic yield of genome-wide CNV testing across different prenatal indications, along with the incremental value of CMA compared with conventional cytogenetic analysis, is summarised in Table 3.

Compared with conventional karyotyping, CMA provides an incremental diagnostic yield of approximately 3–5% by identifying submicroscopic deletions and duplications below the resolution of standard cytogenetic testing. Identification of a pathogenic CNV in this setting often clarifies the underlying aetiology, refines prognostic assessment, and directly informs pregnancy management and recurrence risk counselling [39,66].

Clinically significant CNVs are not confined to pregnancies with ultrasound-detected anomalies. In structurally normal foetuses undergoing invasive prenatal testing for other indications, such as advanced maternal age or high-risk screening results, pathogenic CNVs are identified in approximately 0.4–1.2% of cases [67,68]. These results indicate that the risk of CNVs is not limited to pregnancies with abnormal ultrasound findings. They also support the recommendation that all women undergoing invasive prenatal diagnosis should be offered CMA, regardless of the ultrasound results [41,69].

High-risk pregnancies identified via combined first-trimester screening, maternal serum screening, or NIPT constitute a significant clinical context for invasive genome-wide CNV analysis. Although NIPT demonstrates high sensitivity for common trisomies, it does not reliably detect most pathogenic CNVs or atypical chromosomal abnormalities [70,71]. Evidence from high-risk and anomaly-enriched cohorts suggests that up to approximately 27% of clinically significant chromosomal abnormalities may be missed when reliance is placed on screening approaches alone. Consequently, invasive diagnostic testing incorporating genome-wide CNV analysis remains the recommended pathway for definitive genetic evaluation in high-risk pregnancies [68].

### 5.2. Added Diagnostic Value of Genome-Wide Sequencing Following Non-Diagnostic CMA

In foetuses with structural anomalies and non-diagnostic CMA results, genome-wide sequencing approaches provide additional diagnostic value. Both prospective and retrospective studies show that whole-exome sequencing (WES) and whole-genome sequencing (WGS) improve diagnostic yield by approximately 5–12%, especially in cases with multisystem anomalies or phenotypes suggesting monogenic disorders [72].

By enabling the concurrent detection of CNVs, single nucleotide variants (SNVs), and other classes of structural variation within a single analytical framework, WGS offers the most comprehensive single-assay genomic assessment currently available. Existing evidence supports the consideration of genome-wide sequencing in selected, unresolved prenatal cases following appropriate genetic counselling and multidisciplinary discussion [57,58,60,73,74,75].

## 6. Assessment of Diagnostic Methods

In prenatal genetics, diagnostic decision-making relies on the complementary use of conventional cytogenetic methods and advanced genome-wide approaches. Karyotyping, CMA, and WGS differ not only in analytical resolution and variant detection but also in their clinical role, turnaround time, and suitability for specific prenatal indications. Understanding how these methods perform relative to one another is essential for optimising prenatal diagnostic pathways and ensuring appropriate test selection [4,42,59].

Conventional karyotyping provides a genome-wide overview of chromosomal numbers and large-scale structural organisation and has historically been the cornerstone of invasive prenatal diagnosis. Its principal clinical strength lies in the reliable detection of common aneuploidies and balanced chromosomal rearrangements. However, the limited practical resolution of karyotyping, typically 5–10 Mb, restricts its ability to detect pathogenic submicroscopic CNVs. As a result, diagnostic yield is reduced in many prenatal contexts, particularly in pregnancies complicated by foetal structural anomalies [4,17,20].

CMA has largely addressed these limitations by enabling genome-wide detection of CNVs at substantially higher resolution. CMA consistently demonstrates superior diagnostic yield compared with karyotyping, especially in foetuses with ultrasound-detected anomalies, and has therefore become the preferred first-line genome-wide diagnostic test in invasive prenatal diagnosis [18,37]. Performing CMA on extracted DNA without the need for cell culture further enhances its clinical utility by reducing turnaround time and minimising culture-related failures. At the same time, CMA does not detect balanced chromosomal rearrangements and shows variable sensitivity for mosaicism and triploidy, which may necessitate complementary testing in selected cases [18,40,41,42].

WGS represents the most comprehensive genomic diagnostic approach currently available in prenatal genetics. By combining genome-wide coverage with nucleotide-level resolution, WGS allows for the simultaneous detection of CNVs, SNVs, indels, and a broad range of structural variants [55,58]. This expanded diagnostic scope is particularly valuable in foetuses with complex or multisystem phenotypes and in cases where CMA does not yield a definitive diagnosis. Nevertheless, the routine use of WGS in prenatal settings remains limited by higher costs, increased analytical complexity, and the need for specialised interpretation, ethical oversight, and extensive genetic counselling [56,58,59].

From a practical perspective, differences in laboratory workflow and turnaround time further distinguish these methods. Karyotyping requires viable dividing cells and depends on successful cell culture, which may delay results and complicate time-sensitive clinical decision-making [20]. In contrast, CMA and sequencing-based methods are DNA-based and generally allow for more rapid and flexible workflows [39,50]. While karyotyping remains relatively inexpensive, its limited resolution reduces overall clinical utility in many contemporary prenatal settings. CMA offers a favourable balance between diagnostic yield, interpretability, and cost, whereas WGS currently serves primarily as a second-line or problem-solving test in carefully selected cases.

Taken together, these methods should not be viewed as mutually exclusive but rather as complementary components of a tiered diagnostic strategy. Their relative clinical positioning and practical considerations in prenatal diagnosis are summarised in Table 4.

## 7. International Recommendations and National Context

International professional societies consistently endorse genome-wide CNV analysis as a core component of invasive prenatal genetic diagnosis, particularly in pregnancies complicated by foetal structural anomalies or stillbirths. The central rationale for these recommendations is the superior diagnostic resolution of CMA compared with conventional karyotyping, alongside recognition that sequencing-based approaches have a more selective role in unresolved cases.

Guidelines issued by the American College of Obstetricians and Gynaecologists (ACOG) and the Society for Maternal–Foetal Medicine (SMFM) recommend offering CMA to all patients undergoing invasive prenatal diagnostic procedures. In pregnancies with foetal structural anomalies or stillbirth, CMA is recommended in preference to conventional karyotyping and may replace it as the primary diagnostic test. For structurally normal foetuses undergoing invasive testing, either CMA or karyotyping is considered acceptable, provided patients receive appropriate counselling regarding the relative benefits and limitations of each approach [39,41,69,78].

Similarly, the International Society of Ultrasound in Obstetrics and Gynaecology (ISUOG) recommends CMA as a first-line diagnostic test in pregnancies with foetal structural anomalies. ISUOG guidelines highlight the ability of CMA to achieve an incremental detection rate of approximately 3–5% beyond karyotyping alone [39,79,80,81,82,83]. Given the genetic heterogeneity underlying many foetal malformation patterns, this increase is considered clinically meaningful and supports the routine integration of CMA following invasive sampling.

Major professional societies do not currently recommend WES or WGS for routine prenatal diagnosis. Nevertheless, both ACOG/SMFM and ISUOG acknowledge the clinical utility of sequencing in selected, unresolved cases, particularly when CMA is non-diagnostic and the foetal phenotype suggests a monogenic disorder or multisystem involvement [58,59]. Across all guidelines, a consistent emphasis is placed on the necessity of comprehensive pre-test and post-test genetic counselling. Counselling should explicitly address the possibility of VUS, potential incidental findings, and the implications for pregnancy management [39,41,59,78].

### The Bulgarian Context: Challenges and Disparities

Healthcare systems differ greatly in how genome-wide prenatal diagnostics are implemented. In Bulgaria, access to thorough genome-wide testing remains restricted by structural, financial, and policy-related limitations. Evidence-based prenatal genetic care is continuously challenged by regional differences, varying ultrasound expertise, and a lack of specialised laboratory services [84,85,86,87].

In Bulgaria, genome-wide methods like CMA are typically not reimbursed, and publicly funded testing is mainly limited to targeted analyses for common trisomies [39,52,71,77,80,88]. Consequently, even in high-risk pregnancies, a significant percentage of pathogenic submicroscopic CNVs—including those linked to syndromic disorders and adverse neurodevelopmental outcomes—go undiagnosed. This limited availability contributes to underdiagnosis and diminishes precision in genetic counselling and recurrence risk assessment. For impacted families, the reliance on out-of-pocket testing results in access disparities and limits their ability to make informed reproductive decisions [39,40,71,88,89,90,91].

Alignment with international standards requires the integration of CMA as a first-line diagnostic test, alongside the expansion of national reimbursement schemes. In parallel, the development of standardised national protocols and investment in professional training are necessary to improve equity of access [39,80,88,92,93].

## 8. Prospects, Ethical Issues, and Clinical Impact

By making it possible to identify clinically significant submicroscopic abnormalities, genome-wide CNV analysis has significantly expanded the diagnostic scope of prenatal genetics. CMA and LP-GS demonstrate clear clinical advantages over traditional karyotyping. In pregnancies with ultrasound-detected anomalies and normal karyotypes, genome-wide CNV testing identifies additional relevant findings in approximately 4–10% of cases [40,52,79,88,94].

Significantly, not all pathogenic or probable pathogenic CNVs lead to fully penetrant or distinct genetic disorders. Numerous CNVs exhibit variable expressivity and partial penetrance, or function as susceptibility factors in multifactorial illnesses where genetic predisposition interacts with environmental and other modifying variables. This complexity highlights the necessity for careful interpretation of prenatal CNV results and for thorough, non-directive genetic counselling [39,95,96].

Despite these advantages, genome-wide testing poses important ethical and counselling challenges. Detection of VUS remains a central issue, with reported rates of approximately 1–6% [9,88,97,98]. VUS complicate phenotype prediction and reproductive decision-making, particularly when findings involve susceptibility loci with incomplete penetrance. Incidental or secondary findings, occurring in approximately 1–2% of tests, also necessitate transparent pre-test counselling and non-directive discussion to support parental autonomy [39,89,99].

Future developments are likely to be shaped by the increasing integration of NGS platforms and artificial intelligence–based analytical tools. LP-GS has emerged as a scalable and cost-effective alternative to microarray platforms, particularly in high-throughput environments [53]. For healthcare systems with constrained resources, including Bulgaria, the primary challenge remains equitable access. Ultimately, the integration of CMA and LP-GS into prenatal care represents a fundamental step towards precision perinatal medicine.

## 9. Conclusions

Genome-wide CNV analysis has revolutionised prenatal genetic diagnostics by allowing the accurate identification of submicroscopic abnormalities that exceed the resolution of traditional techniques. Pathogenic CNVs play a significant role in foetal structural anomalies and adverse developmental outcomes, including instances with normal karyotypes.

CMA is presently the most suitable first-line genome-wide diagnostic test for invasive prenatal diagnosis, providing an ideal balance of accuracy, interpretability, and clinical utility. Sequencing-based methodologies provide additional information in specific unresolved cases; however, their broader implementation requires a comprehensive evaluation of interpretative difficulties and counselling resources.

The situation in Bulgaria illustrates the detrimental effects of restricted access to genome-wide diagnostics and emphasises the necessity for policy harmonisation, enhanced professional training, and reform of reimbursement systems. To foster equitable and patient-centred care, it is essential to incorporate genome-wide CNV analysis more thoroughly into national prenatal diagnostic protocols, given its superior diagnostic yield, enhanced clinical utility, and its role in supporting accurate prognostic assessment and informed genetic counselling.

## Figures and Tables

**Table 1:** Conventional cytogenetic methods used in prenatal diagnosis: analytical capabilities and limitations.

Method	Genome Coverage	Practical Resolution	Detectable Abnormalities	Key Advantages	Main Limitations
Karyotyping	Genome-wide	~5–10 Mb	Aneuploidies; large structural CNVs; balanced/unbalanced translocations; inversions; marker chromosomes	Unbiased, genome-wide; detects balanced rearrangements	Low resolution for submicroscopic CNVs; requires cell culture; long turnaround time
High-resolution Karyotyping	Genome-wide	~3–5 Mb	Selected structural abnormalities (prometaphase/prophase)	Improved resolution compared to standard G-banding	Labour-intensive; not routinely available in all settings
FISH (Rapid Aneuploidy)	Targeted	Probe-specific (~100 kb)	Trisomies 13, 18, 21; sex chromosome aneuploidies	Rapid results (24–48 h); does not require cell division	Limited to predefined loci; risk of missing non-targeted anomalies
FISH (Targeted Structural)	Targeted	<100 kb	Known microdeletions, duplications, and cryptic rearrangements	High locus-specific resolution for confirmation	Requires prior diagnostic hypothesis or clinical suspicion

* CNV, copy number variant; FISH, fluorescence in situ hybridisation; kb, kilobase (1000 base pairs); Mb, megabase (1,000,000 base pairs); h, hours.

**Table 2:** Advanced DNA-based technologies in prenatal genetic: analytical scope, strengths, and key limitations.

Technology	Genome Coverage	Main Detectable Variants	Key Strengths	Main Limitations	References
CMA (aCGH/SNP)	Genome-wide	CNVs	First-tier diagnostic test; higher resolution than karyotyping	Does not detect balanced rearrangements; variable sensitivity for mosaicism	[4,39,40,41,42,43,44,45,46,47]
SNP Arrays/Combined	Genome-wide	CNVs; Copy-neutral changes	Detection of AOH/UPD patterns; assessment of consanguinity	Performance depends on probe density and reporting thresholds	[48,49]
LP-GS	Genome-wide	Aneuploidies; CNVs	Scalable NGS-based alternative; performance comparable to CMA	Cannot detect balanced rearrangements; requires validated pipelines	[50,51,52,53]
WES	Coding regions	SNVs; small indels	Useful when monogenic aetiology is suspected; high diagnostic yield	Limited to exonic regions; not for comprehensive structural analysis	[38,54,55,56,57,58,59,60]
WGS	Whole genome	CNVs; SNVs; Indels	Most comprehensive single-assay genomic approach	High complexity of interpretation and counselling	[55,56,57,58,59,60]
NIPT (cfDNA)	Screening only	Common aneuploidies	Non-invasive; high screening performance	Not diagnostic; residual risk in the presence of foetal anomalies	[5,6]

* AOH, absence of heterozygosity; CMA, chromosomal microarray analysis; CNV, copy number variant; cfDNA, cell-free DNA; Indel, small insertion or deletion; LP-GS, low-pass genome sequencing; NGS, next-generation sequencing; NIPT, non-invasive prenatal testing; SNV, single nucleotide variant; UPD, uniparental disomy; WES, whole-exome sequencing; WGS, whole-genome sequencing; SNP, Single nucleotide polymorphism.

**Table 3:** Clinical indications for genome-wide CNV testing in prenatal diagnosis and associated diagnostic yield.

Clinical Scenario	Recommended Diagnostic Approach	Diagnostic Yield of Pathogenic CNVs	Incremental Value	References
Foetal structural anomalies on ultrasound	CMA	~3–13% (most commonly 4–10%)	+3–5% compared with karyotyping	[5,6,39,63,64,65,66,67,68,69,70,71,72,73,74,75,76]
Structurally normal foetuses (invasive testing)	CMA	~0.4–1.2%	Detection of non–age-dependent CNVs	[67,68]
High-risk screening/Positive NIPT	CMA (Invasive)	Variable; residual risk remains	Screening may miss up to ~27% of anomalies	[68,71,77]
Structural anomalies with non-diagnostic CMA	WES/WGS	+5–12% (additional yield)	Identification of monogenic aetiologies	[57,58,72,73,74,75]

* CMA, chromosomal microarray analysis; CNV, copy number variant; NIPT, non-invasive prenatal testing; WES, whole-exome sequencing; WGS, whole-genome sequencing.

**Table 4:** Clinical positioning and practical considerations of diagnostic methods in prenatal genetics.

Method	Typical Role in Prenatal Diagnosis	Key Clinical Strengths	Main Clinical Limitations	References
Karyotyping	Detection of common aneuploidies and balanced rearrangements; complementary testing	Genome-wide overview; identification of balanced chromosomal events	Limited resolution; long turnaround time; low yield for submicroscopic CNVs	[4,17,20]
CMA	First-line genome-wide diagnostic test in invasive prenatal diagnosis	Higher diagnostic yield; ideal for foetal structural anomalies; culture-independent	Does not detect balanced rearrangements; variable sensitivity for mosaicism	[4,39,42,43,44]
WGS	Second-line or problem-solving test following non-diagnostic CMA	Broadest diagnostic scope; simultaneous detection of multiple variant classes	Higher cost; complex interpretation; increased counselling requirements	[55,57,58,59]

* CMA, chromosomal microarray analysis; CNV, copy number variant; WGS, whole-genome sequencing.

## Data Availability

No new data were created or analyzed in this study. Data sharing is not applicable to this article.

## Abbreviations

The following abbreviations are used in this manuscript:

AOH	Absence of heterozygosity
aCGH	Array comparative genomic hybridisation
CMA	Chromosomal microarray analysis
CNV	Copy number variant
cfDNA	Cell-free DNA
FISH	Fluorescence in situ hybridisation
Indel	Small insertion or deletion
kb	Kilobase
LP-GS	Low-pass genome sequencing
Mb	Megabase
NGS	Next-generation sequencing
NIPT	Non-invasive prenatal testing
SNP	Single nucleotide polymorphism
SNV	Single nucleotide variant
UPD	Uniparental disomy
WES	Whole-exome sequencing
WGS	Whole-genome sequencing
